# Potential effect of non-thermal plasma for the inhibition of scar formation: a preliminary report

**DOI:** 10.1038/s41598-020-57703-6

**Published:** 2020-01-23

**Authors:** Xiao-Feng Wang, Qing-Qing Fang, Bing Jia, Yan-Yan Hu, Zheng-Cai Wang, Ke-ping Yan, Sheng-Yong Yin, Zhen Liu, Wei-Qiang Tan

**Affiliations:** 1 Department of Plastic Surgery, Sir Run Run Shaw Hospital, Zhejiang University School of Medicine, Hangzhou Zhejiang Province, P.R. China; 2 Institute of Industrial Ecology and Environment, Collage of Chemical and Biological Engineering, Zhejiang University, Zhejiang Province, P.R. China; 3 Key Laboratory of Combined Multi-organ Transplantation, Ministry of Public Health, The First Affiliated Hospital, Zhejiang University School of Medicine, Hangzhou Zhejiang Province, P.R. China; 4 Department of Plastic Surgery, The Fourth Affiliated Hospital, Zhejiang University School of Medicine, Yiwu Zhejiang Province, P.R. China

**Keywords:** Outcomes research, Skin diseases

## Abstract

Non-thermal plasma (NTP) is a promising biomedical tool for application to wound healing. However, there is limited scientific evidence that confirms its efficacy to inhibit scar formation. This study aims to investigate the role of non-thermal plasma in scar formation. Two full-thickness dorsal cutaneous wounds of rats were treated with either a non-thermal helium plasma jet or helium. It was determined that the non-thermal plasma jet accelerated the wound healing process from 5 days after surgery (day 5: 41.27% ± 2.351 vs 54.7% ± 5.314, p < 0.05; day 7: 56.05% ± 1.881 vs 75.28% ± 3.914, p < 0.01; day 14: 89.85% ± 2.991 vs 98.07% ± 0.839, p < 0.05). The width of the scars for the NTP group was narrower than those of control group (4.607 ± 0.416 mm vs 3.260 ± 0.333 mm, p < 0.05). In addition, a lower level of TGF-β1, p-Smad2 and p-Smad3 were detected in the NTP treated wounds (p < 0.05, p < 0.01 and p < 0.01). As expected, α-SMA was also significantly decreased in the NTP treatment group (p < 0.01). Moreover, the expression of type I collagen and the proportion of type I to III collagen were lower in the NTP group (p < 0.05). The results of the study suggest that NTP may play a potential role in scar formation by inhibiting the TGF β1 signal pathway and reducing the levels of α-SMA and type I collagen, and may have clinical utility in the future.

## Introduction

Scar formation is an inevitable outcome after physical, biological, and chemical injury of the skin, The phenomenon is characterized by excessive deposition and irregular distribution of extracellular matrices (ECM), in addition to an overproduction of fibroblasts^1–3^. Patients with severe scars caused by burns, scalds or serious traumas, experience physical and mental anguish that is typically associated with the dysfunction and disfigurement caused by tissue hypertrophy or severe contraction^4^. Therefore, even incremental improvements in scar management could result in significant benefits to patients. To date, numerous therapeutic approaches have been developed for the treatment of scars including surgical excision, corticosteroid injection, and laser therapy^5,6^. However, in many cases these treatments do not result in satisfactory outcomes. The treatment of scars is still a formidable task, and advanced treatments or techniques for the minimization of scarring are needed.

Plasma medicine, a rapidly developing interdisciplinary field, has already developed as a new innovative approach for biomedical and clinical applications^7^. Emerging evidence suggests that non-thermal plasma (NTP) is potentially beneficial for bacteria disinfection, blood coagulation, and cancer therapy^8–12^. NTP has also been shown to play a role in wound healing^13^. However, there are limited experimental studies on the application of NTP to inhibit scar formation. In this study, we aimed to investigate the efficacy of non-thermal plasma in the inhibition of scar formation in a rat model. Based on histological observation and immunohistochemistry quantitative analysis, we concluded that plasma exposure can effectively inhibit scar formation and may have clinical application in the future for scar treatment.

## Results

### Non-thermal plasma jet did not cause thermal damage

**Figure 1:**
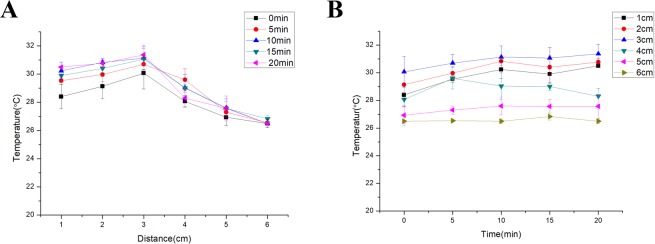
The non-thermal plasma (NTP) jet did not cause thermal damage. (**A**) Relationship between the temperature of the non-thermal plasma jet, and the distance from the nozzle of the non-thermal plasma gun at different times. (**B**) Relationship between the temperature of the non-thermal plasma jet and time at different distances from the nozzle of the non-thermal plasma gun.

To avoid thermal damage, we conducted an experiment on the change in the temperature of the non-thermal plasma jet with distance from the nozzle of the non-thermal plasma gun prior to animal study. The results revealed that the temperature at 3 cm from the nozzle of the plasma jet was the highest, and the maximum temperature did not exceed 32 °C (Fig. 1A). Considering that the amount of heat generated by the non-thermal plasma gun increased with time, we also monitored the temperature change as a function of time. The data revealed that for a given position, the temperature did not change significantly with time (Fig. 1B).

### Non-thermal plasma jet accelerated wound closure

**Figure 2:**
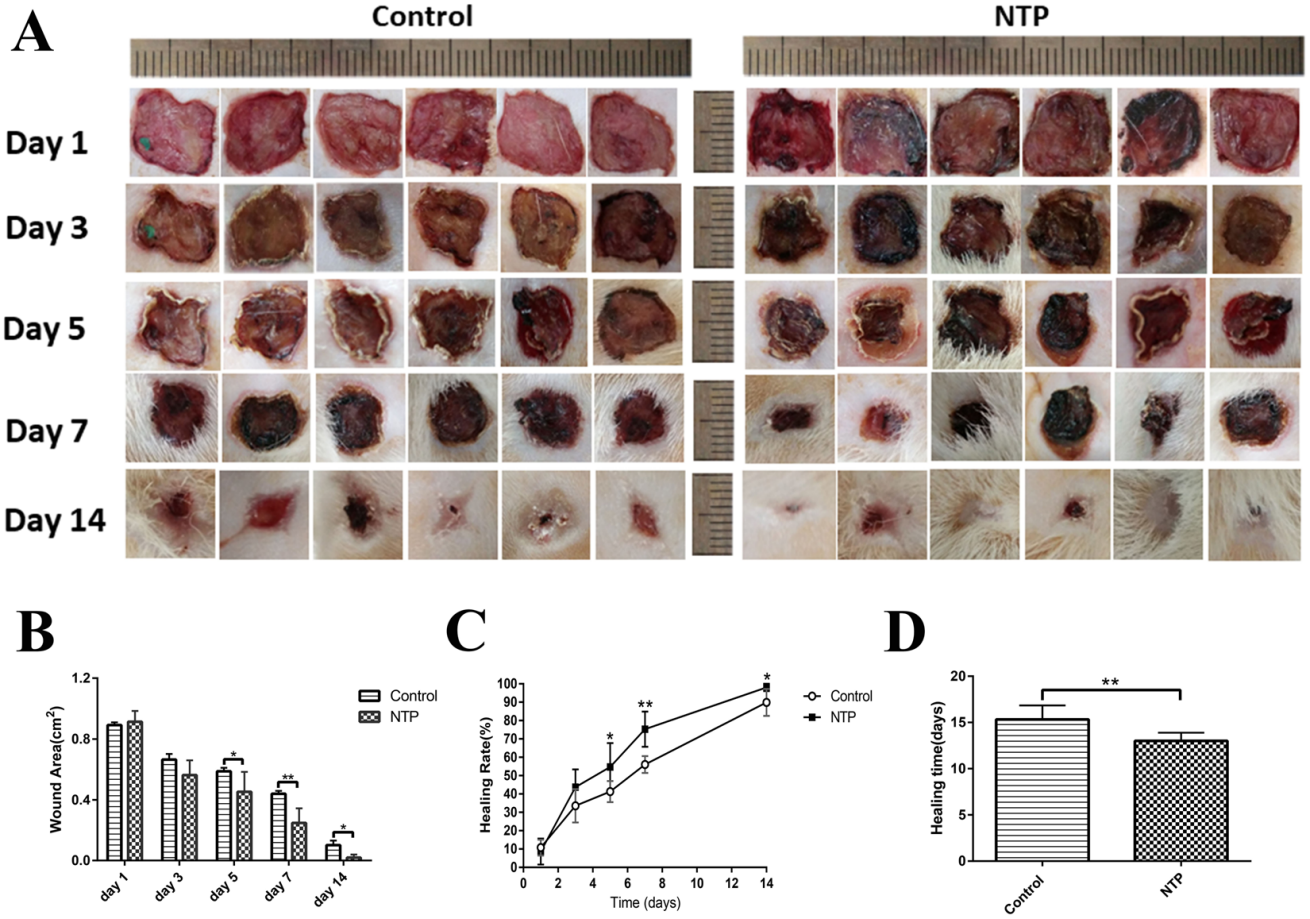
Non-thermal plasma (NTP) jet accelerated wound closure. *p < 0.05, **p < 0.01. (**A**) Images of the skin wounds in control and NTP treated groups on days 1, 3, 5, 7 and 14 after surgery. Scale bar = 1 cm. (**B**) Statistical analysis of wound area in 2 groups on days 1, 3, 5, 7, 14 after surgery. The groups are the control group and the NTP treated group (n = 12 wounds in 6 rats). (**C**) Statistical analysis of wound healing rate in these two groups. (**D**) Healing times in the control and NTP treated groups.

The wound closure for several wounds was evaluated on days 1, 3, 5, 7, 14 by determining the unclosed wound area (Fig. 2A). It is evident that there were blood clots (“black area”) on day 1 for the NTP treated wounds, and the effect of promoting blood coagulation has been confirmed in previous reports. Although there was no difference in wound closure among the NTP and control groups in the early phase of the healing process (0.892 ± 0.018 cm^2^ vs 0.914 ± 0.029 cm^2^ and 0.665 ± 0.037 cm^2^ vs 0.563 ± 0.040 cm^2^ on day 1 and 3 after surgery), the NTP treatment group exhibited a significant improvement in wound closure on day 5, 7 and 14 (0.587 ± 0.024 cm^2^ vs 0.453 ± 0.053 cm^2^, p < 0.05; 0.440 ± 0.019 cm^2^ vs 0.247 ± 0.039 cm^2^, p < 0.01; 0.102 ± 0.030 cm^2^ vs 0.019 ± 0.039 cm^2^, p < 0.05), compared to the control group (Fig. 2B). The non-thermal plasma treatment drastically accelerated the wound healing process from 5 days after surgery (day 5: 41.27% ± 2.351 vs 54.7% ± 5.314, p < 0.05; day 7: 56.05% ± 1.881 vs 75.28% ± 3.914, p < 0.01; day 14: 89.85% ± 2.991 vs 98.07% ± 0.839, p < 0.05; Fig. 2C). Moreover, most of the wounds of the NTP group were closed 14 days after the initial wounding, whereas the wounds of control group closed at a later time (p < 0.05, 13 ± 0.8944 days vs 15.33 ± 1.506 days, Fig. 2D). These data suggest that non-thermal plasma can promote wound healing and enhance the wound healing rate in rats with acute skin wounds.

### Non-thermal plasma inhibited scar formation *in vivo*

**Figure 3:**
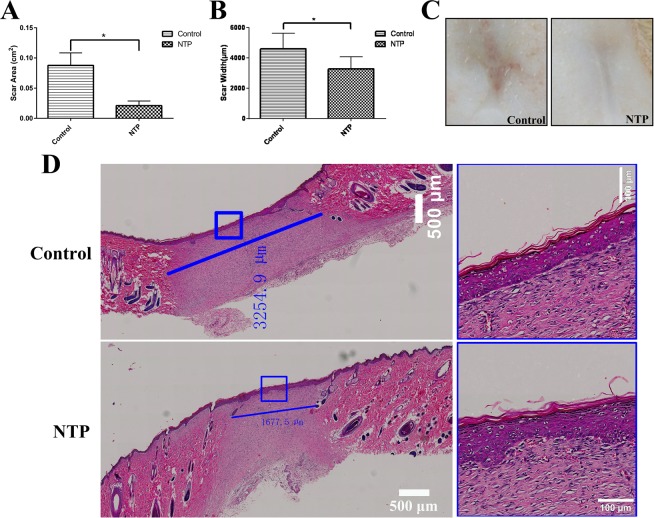
Non-thermal plasma inhibited scar formation. *p < 0.05 (**A**) Statistical analysis of scar area on day 21 after surgery in the control and NTP treated groups (based on images acquired on day 21 postoperatively). (**B**) Statistical analysis of the scar width for each group was performed (based on scanning images of HE stained specimens). (**C**) Representative rat scar on day 21 postoperatively in the control and the NTP treated groups. (**D**) Typical HE stained sections of rat scar tissue harvested on day 21 after surgery. The blue lines are used to determine the scale of the scar, the white bar = 500 μm. The image on the right represent a magnified view of the blue rectangle in the left image, the white bar = 100 μm.

The scar area was measured on day 21 after surgery. In the treatment group, this area was smaller and less remarkable compared to the control group (Fig. 3A,C). After all the wounds of these two groups were completely epithelialized on day 21, the animals were sacrificed and scar tissues were harvested. H&E (Histological Examination) staining revealed that the scar width in the plasma group was not only smaller (4.607 ± 0.416 mm vs 3.260 ± 0.333 mm, p < 0.05), but the scars were also better re-epithelialization compared to the control group (Fig. 3B,D). These data revealed a smaller and aesthetically acceptable scar in the NTP treatment group.

### NTP down-regulated the expression of TGF-β1 and the phosphorylation of Smad2/3

**Figure 4:**
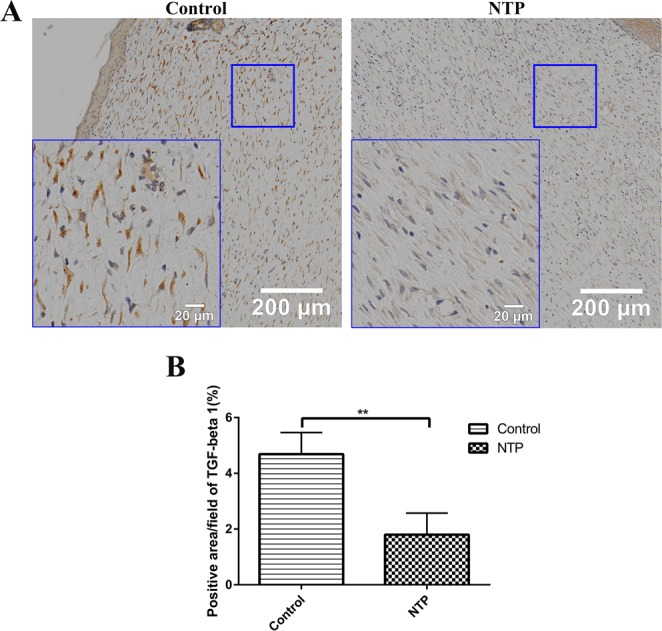
Non-thermal plasma down-regulated the expression of TGF-β1. **p < 0.01. (**A**) Typical images of TGF-β1 for immunohistochemical staining in the control and NTP groups. The image on the lower left corner represent a magnified view of the blue square. Bar = 200 μm/20 μm. (**B**) Statistical analysis of the density of TGF-β on day 21 in the control and the NTP treated wounds.

**Figure 5:**
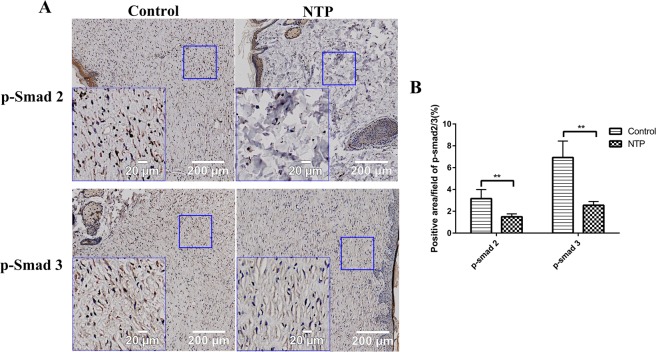
Non-thermal plasma down-regulated the expression of p-Smad 2/3. **p < 0.01. (**A**) Typical images of p-Smad 2 and p-Smad 3 after immunohistochemical staining in the control and NTP groups. The image in the lower left corner is a magnified view of the blue square. Bar = 200 μm/20 μm. (**B**) Statistical analysis of p-Smad2/3 on day 21 in the control and the NTP treated wounds.

The TGF-β1/Smad2/3 pathway is considered as one of the most important signaling pathways in scar formation. To further investigate the underlying mechanism of NTP for the inhibition of scar formation, we used immunohistochemistry staining to quantify TGF-β1 and smad2/3 expression. The immunohistochemistry staining analysis of the tissue samples revealed that the expression of TGF-β1 in the treatment group was significantly lower compared to the control group (p < 0.05, Fig. 4A,B). Phosphorylated Smad2 and Smad3 (p-Smad2 and p-Smad3), the biologically active form of Smad2 and Smad3 protein, as expected, also significantly decreased in the NTP treatment group (p < 0.01, Fig. 5A,B).

### NTP suppressed α-SMA expression and collagen levels

**Figure 6:**
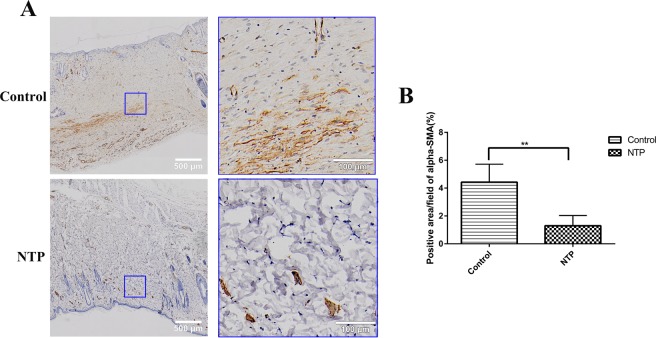
The non-thermal plasma jet decreased α-SMA expression. **p < 0.01. (**A**) Typical images of α-SMA after immunohistochemical staining for the control and the NTP groups. The image on the right is a magnified view of the blue square in the left image. Bar = 500 μm/100 μm. (**B**) Statistical analysis of the density of α-SMA on day 21 in the control and the NTP treated wounds.

**Figure 7:**
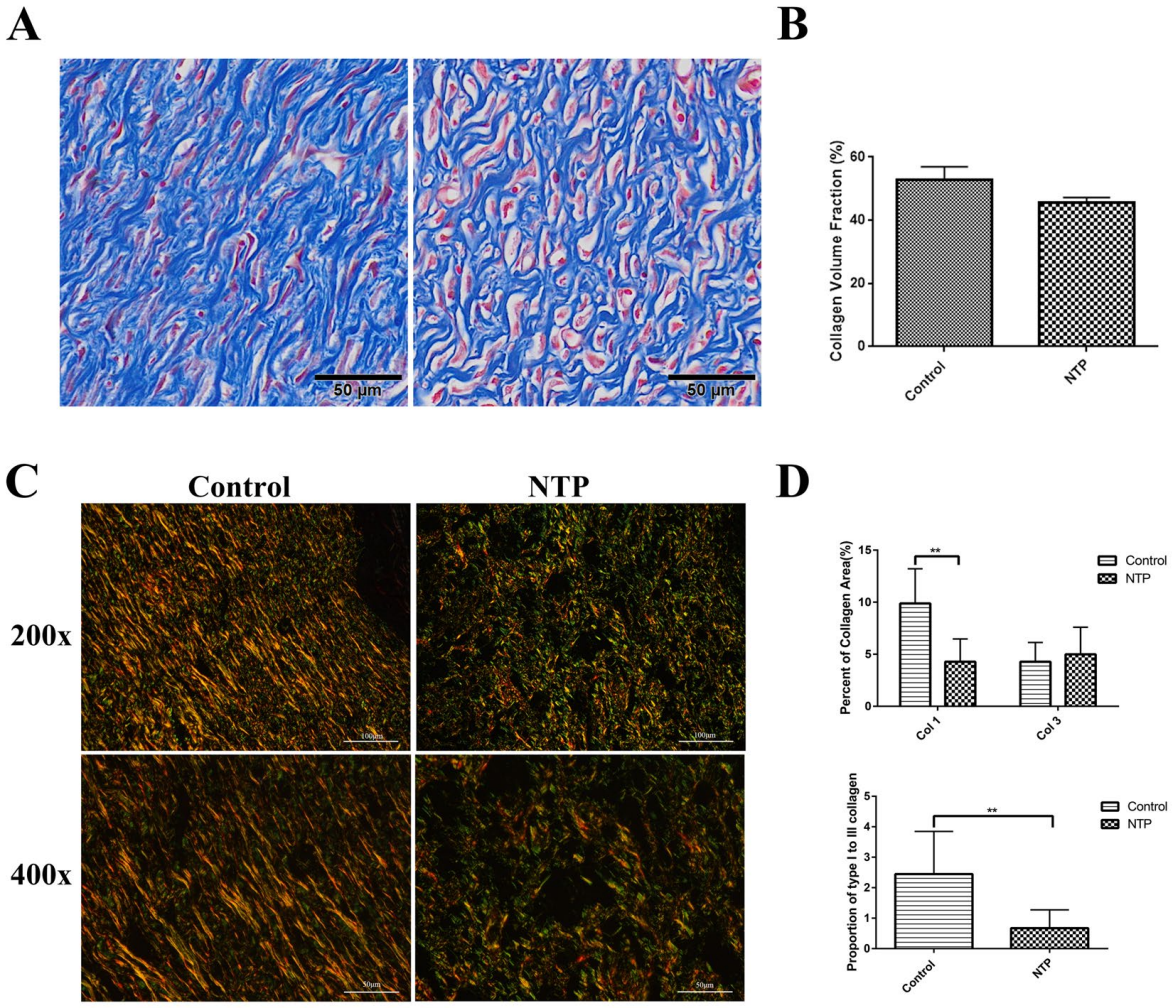
The non-thermal plasma jet decreased collagen levels. **p < 0.01. (**A**) Typical images of Masson’s trichrome staining for the control and the NTP groups. Bar = 50 μm. (**B**) Statistical analysis of the collagen volume fraction in the control and NTP groups. (**C**)Typical images of Sirius red staining using a polarizing microscope for magnifications of 200x and 400x for the control and the NTP treated wounds. Bar = 100 μm (200x) and 50 μm (400x). (**D**) Statistical analysis of the percent area of type I, type III collagen and the proportion of type I to type III collagen.

It has been demonstrated in previous studies that the expression of alpha-smooth muscle actin (α-SMA) positive myofibroblasts and collagen is higher in various pathological scars, which is closely related to scar formation. Therefore, to determine whether NTP treatment suppresses scar information by affecting the expression of α-SMA and collagen, immunohistochemistry, Masson’s trichrome staining, and Sirius red staining assay were performed. The results revealed that the quantity of α-SMA was lower in the NTP treatment group than the control group (p < 0.01, Fig. 6A,B). Masson’s trichrome staining revealed prominent collagen deposition (blue) in the control group, whereas collagen deposition was decreased in the NTP treated wound tissues (Fig. 7A,B). In addition, the Sirius red staining indicated that the scar tissue in the NTP treatment group was loosely arranged with less collagen, whereas there was more collagen in the untreated control group (Fig. 7C). Quantitative analysis of type I and type III collagen was performed using a polarizing microscope. Collagen I was downregulated in the scar tissue of the NTP treatment wounds (p < 0.05). The level of type III was a little higher in the experimental group (p > 0.05). Although there was no significant difference in the levels of collagen III among these two groups, the ratio of collagen type I to type III decreased significantly in the NTP treatment group (p < 0.05, Fig. 7D). These data demonstrated that NTP reduced the levels of α-SMA and type I collagen, and was therefore, a potentially effective therapy for scar management.

## Discussion

Skin is the largest organ of the human body and is an anatomical barrier for pathogens and damage between the internal and external wound environment in bodily defense^14^. Intact skin is essential for the survival of an organism, and thus wound healing is a vital process^15^. Wound healing is a complicated process in which skin, and the underlying tissues are repaired after injury^16^. This process consists of four stages: hemostasis, inflammation, proliferation, and tissue remodeling^17^. However, given that we lack the ability to perform complete regeneration, the outcome of wounding healing is always scar formation in adult mammals, including humans. A scar is characterized by excessive deposition and irregular distribution of ECM, and the overproduction of fibroblast. It can result in a series of physiological and physiological symptoms, thereby reducing the quality of life of affected individuals.

The therapeutic approaches for scar management can be classified in two main categories: conservation (laser therapy, topical silicone gel, compression therapy) and invasive (surgery, steroid injections) therapies^18^. Although there are numerous therapeutic approaches, many of these treatments may serve as a placebo. As such, the therapeutic effect of current methods is still unsatisfactory.

Non-thermal plasma (NTP) is an ionized state of matter that is similar to a gas. Based on its antimicrobial qualities, it is widely used in industry and medical fields. Its usage includes sterilization of fruits, vegetables and surgical instruments, blood coagulation, and cancer therapy^8–12^. Non-thermal plasma appears to be a promising biomedical tool in wound healing, and the existing body of work suggests that it could promote healing in acute and chronic skin wounds^7,19^. Although many researchers have started to investigate NTP in wound healing, there are few experimental studies on its effect on scar formation, which is the result of wound healing.

It has been proved that non-thermal plasma does not cause DNA damage^20^ or any thermal damage to articles^21^. Proteins tend to denature at temperatures above 40–45 °C. When the temperature is higher than 45 °C, proteins are irreversibly damaged. DNA and RNA are also potential macromolecular targets of thermal injury; however, they are typically only damaged above 85–90 °C^22^. To avoid thermal damage or protein denaturation, we conducted an experiment to investigate the change in temperature of the non-thermal plasma jet with distance from the nozzle of the gun prior to the animal study. The results revealed that the maximum temperature was 32 °C, regardless of the distance or exposure times. In our study, the non-thermal plasma jet is 10 cm in the maximum length. When applied to wounds, the wound was about 3–4 cm away from the nozzle of the non-thermal plasma gun. The temperature at these distances did not cause thermal damage or protein denaturation and therefore, did not result in damage to the animal tissue.

To confirm the results of previously published research on wound healing, we first observed the efficacy of NTP on the healing in an acute rat wound model. On day 1, compared to the control group, we found that there were black blood clots (“black area” in Fig. 2A) in the NTP treatment wounds. Similar to the results in previous studies^23^, we found that non-thermal plasma could actually promote blood coagulation. Moreover, compared to the control group, the wound area in the NTP treatment group was significantly smaller on days 5, 7 and 14. The time for wound healing in the treatment group was 2 days shorter compared to the control group. The data proved that NTP indeed promotes the healing of wounds, as shown in previously published reports. In addition, H&E staining of the scar sample on day 21 reveled that the scar width for the NTP group was not only smaller, but also superior re-epithelialization than the control group. To clarify the underlying mechanism, immunohistochemistry quantitative analysis, and staining approaches to detect and estimate collagen levels were performed for further analysis of the effect of NTP on the inhibiting of scar formation.

Transforming growth factor-β (TGF-β) is a secreted cytokine that plays a prominent role in many cellular signaling pathways, including proliferation, migration, adhesion and differentiation^24^. As an upper reaching signal molecule, TGF-β regulates the expression of multiple downstream signaling molecules involved in scar formation via both canonical and noncanonical pathways^25^. The TGF-β1/Smad2/3 pathway is considered as one of the most important signaling pathways in scar formation because it supports the overproduction of ECM components and the over-proliferation of fibroblasts, which was confirmed in our previous study^26,27^. Thus, down-regulating the expression of the TGF-β1/Smad2/3 pathway is a promising strategy in scar management. In our study, the immunohistochemistry analysis of TGF-β1, p-Smad2, and p-Smad3 proved that NTP really suppressed the expression of TGF-β1 and p-Smad2/3.

On a molecular level, TGF-β induces the transformation of fibroblasts to myofibroblasts, which is a key step in all fibrotic processes^28^. The transdifferentiating fibroblasts are then subjected to various types of mechanical forces during phonation and vibration may stimulate α-SMA expression in response to tension^29^. α-SMA is a widely accepted marker of myofibroblast differentiation, which is responsible for contraction during wound healing as a result of its stress fibers^30–32^. An increasing number of studies have demonstrated that the expression of α-SMA is higher in various pathological scars and is essential to scar formation after injury^33^. At normal levels, α-SMA positive myofibroblasts can synthesize and secrete large amounts of collagen, growth factors and enzymes, which can promote wound healing^34^, but usually disappears at the later stages of this process^35^. However, the continued presence and activation of α-SMA, which promotes the maturation of myofibroblasts, would result in an increase in the deposition of ECM proteins, which results in tissue fibrosis^36–38^. In our study, the results revealed that the quantity of α-SMA was lower in the NTP treatment group compared to the control group. Therefore, to prevent tissue fibrosis, NTP is effective in blocking the expression of α-SMA in scar formation.

Instead of the regular collagen fibers found in normal tissue, scar tissue is composed of irregularly arranged collagen. During the remodeling phase, type III collagen, which is prevalent during proliferation, is replaced by type I collagen which enhances the tensile strength of the wound^39^. However, excessive type I collagen leads to pronounced scar formation. Previous studies have shown that the reduction the levels of collagen I is an effective way of inhibiting scar formation^40^. In our study, Masson’s trichrome staining, which cannot distinguish between type I and type III collagen, revealed that the total collagen decreased after NTP treatment. Moreover, Sirius red staining reveled that type I collagen was downregulated after NTP treatment. Although there was no difference in the levels of type III collagen among the two experimental groups, the ratio of collagen type I and type III was significantly lower in the NTP treatment group. The data indicated that NTP decreased the levels of type I collagen, which is another possible reason for the reduction in scar formation.

The data may imply that there is a correlation between NTP and scar formation. However, due to the limited sample size of the study, the results obtained for histological examination and immunohistochemical staining are not sufficient to verify the specific effect of this technique on scar formation. There is no firm evidence regarding the specific mechanisms of NTP in the inhibition of scar formation. It is still a question that requires further reflection and inquiry to determine how NTP regulates the aforementioned pathways.

In conclusion, we found that non-thermal plasma jet not only accelerated wound healing, but also inhibited the formation of scar tissue. It may play an anti-fibrotic role in scars by reducing the TGF β1/Smad2/Smad3 signal pathway and regulating the levels of α-SMA and type I collagen. This study may provide a new perspective on scar treatment.

## Materials and Methods

### Physicochemical characterization of the plasma

**Figure 8:**
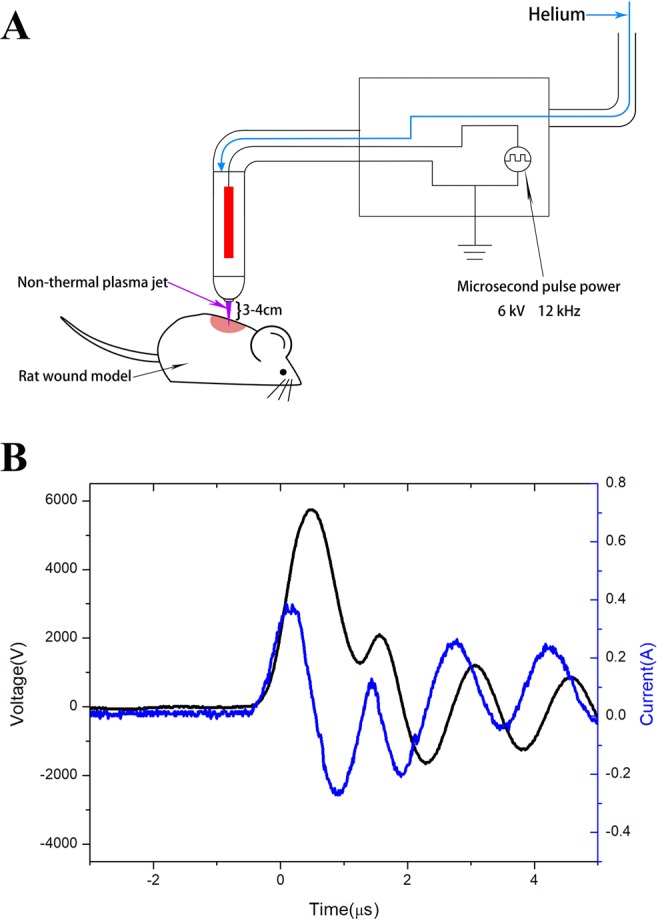
Physicochemical characterization of the plasma. (**A**) Schematic representation of the non-thermal plasma generator. (**B**) The electrical parameters of the non-thermal plasma jet.

In the experiment, we used a custom-built plasma jet, which has been reported in our previous work (Fig. 8A,B)^41^. The plasma device consists of a microsecond pulsed power supply and a handheld jet gun. The pulse power source can drive the plasma jet reactor. In this experiment, the pulse width was 1–2 µs, the peak voltage was 6 kV, and the repetition rate was 12 kHz. The average plasma power maintained at less than 10 W, to avoid potential damage to the animal tissue. Helium gas was used in the experiment, at a flow rate of 8 L/min. The diameter of the handheld jet gun was approximately 25 mm, while the diameter of the plasma jet was approximately 4 mm with a maximum length of 10 cm. When applied to the wounds, the wound was approximately 3–4 cm away from the nozzle of the non-thermal plasma gun.

### Animals

Six male Sprague Dawley rats (8–14 weeks) with body weights in the range of 225–240 g were used in the study (Laboratory Animal Center of Zhejiang Academy of Medical Sciences, Hangzhou, China). Randomization was performed by an independent central statistical unit. All animal experiments were approved by the Zhejiang University Animal Care Committee. The experimental procedures and animal maintenance were performed in accordance with the guidelines for animal experiments of the Institutional Animal Care and Use Committee of Zhejiang University. All surgical operations were performed under air anesthesia(isoflurane).

### Wound healing study

The rats were anesthetized using 2% isoflurane and the hair on their backs was removed using an electric shaver and depilatory cream. Two 1 × 1 cm^2^ full-thickness skin (including the dartos) parallel to but 2 cm away from the midline, was excised on both sides of the dorsal skin. After wound generation, the left wounds were a treated with a non-thermal helium plasma jet for 1 min (every 5 minutes) for a total of fourth daily plasma treatments. The right wounds were exposed to helium as a control group, until the wound healed. Images of the wounds were acquired on days 1, 3, 5, 7, 14, 21 after surgery. The surface area of the wounds was grossly measured and the relative wound area was calculated using Image J software (National Institutes of Health, USA). The healing rate of the wound was calculated according to the following formula:

$$\frac{{\rm{area}}\,{\rm{of}}\,{\rm{original}}\,{\rm{wound}}-{\rm{area}}\,{\rm{of}}\,{\rm{measured}}\,{\rm{wound}}}{{\rm{area}}\,{\rm{of}}\,{\rm{original}}\,{\rm{wound}}\,}\times 100 \% $$

### Histological examination

On day 21, the rats were anesthetized via isoflurane inhalation, and the scars tissue include 5 mm unwounded skin were collected. The scar samples were fixed overnight at 4 °C in 4% paraformaldehyde (in 0.1 M PBS, pH 7.4). The tissues were dehydrated with a graded series of ethanol and butanol, then embedded in paraffin blocks. Tissue sections of 4 μm were stained with H&E or Sirius red staining and Masson’s trichrome staining. Using an Olympus vs120 Virtual Slide Microscope, images of the H&E stained specimens were scanned using a 10× objective and the images of the Masson’s trichrome stained samples were obtained using a 20× objective. To quantitative analysis the deposition of type I and type III collagen, images were acquired of Sirius red stained specimens using a polarizing microscope (Nikon, TKY, Japan). The scar width on day 21 and the results of immunohistochemistry were calculated using the Image-Pro Premier 3D software (Media Cybernetics, MD, USA). Quantitative analysis of type I and type III collagen was performed using the Image-Pro Plus 6.0 software (Media Cybernetics, MD, USA).

### Immunohistochemical staining

To analysis the expression of TGF-β1, p-Smad2/3,and α-SMA, rabbit polyclonal antibody directed against TGF-β1 (1:300, Servicebio, Wuhan, China), p-Smad2(1: 100, Affinity Biosciences, OH, USA), p-Smad3(1: 100, Affinity Biosciences, OH, USA), α-SMA (1: 2000, Servicebio, Wuhan, China), and HRP-conjugated goat anti-rabbit IgG (1: 200, Servicebio, Wuhan, China) secondary antibody were used. The sections were initially hydrated using a graded ethanol series, then incubated with 3% H_2_O_2_ for 10 min. To recover antigens, these sections were soaked in 10 Mm citrate buffer solution and heated twice in a microwave oven. After cooling down to room temperature, the slides were then thoroughly washed with PBS and blocked with 3% BSA for 30 min. Sections were incubated with primary antibodies overnight at 4 °C. The following day, the slides were washed with PBS and then incubated for 50 min at room temperature with HRP-conjugated goat anti-rabbit IgG secondary antibody. After washing for three times with PBS, the staining was visualized after incubation with a DAB-H_2_O_2_ solution for 5 min, followed by hematoxylin for 3 min, dehydrated with ethanol, and sealed in resinene for microscopic observation. The images were scanned at 20× magnification using an Olympus vs120 Virtual Slide Microscope. The positive cells in the wound area were identified using the Image-Pro Premier 3D software (Media Cybernetics, MD, USA).

### Statistical analysis

Data were expressed as mean ± Standard Deviation (SD) and calculated by GraphPad Prism Software (GraphPad Software, CA, USA). The statistical significance of differences between control wounds and non-thermal helium plasma jet treated wounds was analyzed using Student’s *t* test. *p*-Values less than 0.05 were considered statistically significant.
